# Half-Timers

**Published:** 1902-09-20

**Authors:** 


### HOSPITAL CLINICS AND MEDICAL PROGRESS.

### "HALF-TIMERS."

A VERY remarkable paper was read before the British Medical Association, at Manchester, by Dr. Alexander Campbell, certifying factory-surgeon of Dundee, in which he dealt with, and gave the highest praise to, the system of half-time employment and half-time education. The half-timer, he says, may be taught either on the alternate days or on the half-day system, but those who have had experience of both systems agree that the half-day system is the better and more natural one, a short lesson every day giving results far superior to a lengthy lesson every second day. In the largest school in Dundee under the School Board in which both day and half-time scholars are taught, there are 450 day scholars and 158 half-time pupils. Amongst the day scholars only 15 have reached class V., and the average age of these is 13. In the half-time department of this school 60 have reached class VI., 60 class V., and the remainder class IV. Curiously enough, even under the alternate-day system which is adopted by all Board Schools in Dundee, the average age in class V. of this department is exactly the same—that is 13, as in the full-day department. The half-time schools in connection with separate works are solely for the education of children belonging to each special factory. They are all worked on the half-day system, the children being in school each alternate week from 9 A.M. to 11.45 A.M., and from 12.15 P.M. to 3 P.M., two hours and three-quarters each attendance. These schools are generously staffed and have almost invariably gained the "excellent" grant from the inspector, and have kept side by side with the best day schools. No home-lessons are given, but the work done is exactly similar to that given in the same classes at day schools. The short hours prevent brain weariness, and the absence of home-lessons prevents jaded minds. Girls coming from the Sixth class of ordinary day schools cannot keep step with the Fifth class girls trained under this system. Experience shows that manual labour quickens the faculties and the rigidly enforced regular attendance prevents lagging, for there are no truants at half-time schools. The children know that they cannot work unless they go to school. Dr. Campbell says, "I have never known a child to be injured in health by working half-time. They always show to advantage when physically compared with day scholars of the same age. Indeed, the physical health of the half-timer is demonstrated by the fact that at our largest half-time school, in the works of Messrs. Baxter Brothers, they make an average attendance of 98·5 per cent., and if the few cases of measles, &c., are taken into account the attendance is practically 100 per cent., whereas amongst day scholars in the Board schools the average attendance is 85 per cent." A result is that the early habits of industry, the discipline, the training to work at an early age, has created a very fine type of factory worker. The puny feeble half-timer is, under the half-day system, an unknown quantity. "The fact is that white-faced, delicate-looking children attending school the whole day improve physically and mentally from the day they enter the factories and become half-timers. They are better fed, and are taught habits of industry and regularity, and the short hours, change of occupation, with afternoons and evenings free for necessary playtime, conduce to rapid growth." It has been said that half-time boys drift into mill life, and are never fit for anything else, but so far from this being the case only a very few of them are required as mill hands. Indeed, half-time boys advancing to full time are much inquired for, and are preferred to lads direct from school as apprentices, and as a rule succeed better. Dr. Campbell's recipe for many educational difficulties would be to raise the full-time age to 15 years, and he says that were this the law half-time schools would be universal. When a girl became 15 years of age, instead of being turned adrift, unable to earn a living, "she would at once earn the wages of a woman, having practically served an apprenticeship, and having at the same time received a sound education."

